# Genomic and phenotypic characterization of a *Clostridioides difficile* strain of the epidemic ST37 type from China

**DOI:** 10.3389/fcimb.2024.1412408

**Published:** 2024-10-18

**Authors:** Chunhui Li, Joshua Heuler, Duolong Zhu, Xiujuan Meng, Soumyadeep Chakraborty, Céline Harmanus, Shaohui Wang, Zhong Peng, Wiep Klaas Smits, Anhua Wu, Xingmin Sun

**Affiliations:** ^1^ Department of Infection Control Center of Xiangya Hospital, Central South University, Changsha, China; ^2^ Department of Molecular Medicine, Morsani College of Medicine, University of South Florida, Tampa, FL, United States; ^3^ Department of Medical Microbiology, Leiden University Medical Center, Leiden, Netherlands

**Keywords:** *Clostridioides difficile*, pathogenicity locus (PaLoc), ST37, RT017, virulence, toxin

## Abstract

*Clostridioides difficile* strains of sequence type (ST) 37, primarily including PCR ribotype (RT) 017, are prevalent in mainland China. Our study aimed to compare the major virulence factors of an epidemic *C. difficile* isolate of ST37 type (Xy06) from China with the well-characterized *C. difficile* reference strains R20291 (RT027) and CD630E (ST54), as well as a Chinese ST54 strain (Xy07) isolated from the same hospital. The Xy06 genome was predicted to harbor two complete prophages and several transposon-like elements. Comparative analysis of PaLoc revealed a truncated tcdA gene, a functional tcdB gene, a functional tcdC gene, and well-conserved tcdR and tcdE genes. Phenotypic comparisons showed that Xy06 was a robust producer of TcdB, readily sporulated and germinated, and strongly bound to human gut epithelial cells. In a mouse model of *C. difficile* infection, Xy06 was more virulent than strains CD630E and Xy07 and was comparable to strain R20291 in virulence. Our data suggest the potential threat of the epidemic ST37 strains in China.

## 1 Introduction

The symptoms of *Clostridioides difficile* infection (CDI) are mainly caused by toxin A (TcdA) and toxin B (TcdB), which are encoded by the *tcdA* and *tcdB* genes, respectively, in the pathogenicity locus (PaLoc). The PaLoc also contains three other genes (*tcdC, tcdE*, and *tcdR*) implicated in toxin expression or release (Martin-Verstraete et al., 2016). Besides TcdA and TcdB, certain strains, including the epidemic RT027 strains, also express the binary toxin (CDT), which is encoded on a different locus (CdtLoc). A small percentage of *C. difficile* strains produce a functional TcdB and a truncated, nonfunctional TcdA. These TcdA-negative, TcdB-positive (A-B+) strains are mostly typed as ST37/RT017 with few exceptions (Imwattana et al., 2019, Imwattana et al., 2022). *C. difficile* RT017 ranks among the most successful RTs of *C. difficile* in the world (Imwattana et al., 2019, Imwattana et al., 2022). Although *C. difficile* RT017 appears to originate from Asia, it has spread globally and caused multiple outbreaks worldwide (Cairns et al., 2017). Several publications reported that infections with strains of type ST37/RT017 are the leading epidemic healthcare-associated CDIs in China (Yan et al., 2013; Jin et al., 2017; Yang et al., 2017) and have attracted increasing attention (Hawkey et al., 2013; Du et al., 2014). Interestingly, ST81 (A-B+) strains have been reported to be dominant in some hospitals in Shanghai and Beijing in China. ST81 strains are genetically close to ST37 but produce less TcdB than ST37 strains (Senoh et al., 2015; Wang et al., 2018a; Yang et al., 2020; Su et al., 2021b; Wu et al., 2022). Several studies suggest that *C. difficile* RT017 can cause clinical disease symptoms that are indistinguishable from those caused by RTs of *C. difficile* strains producing both TcdA and TcdB (A+B+) (Goorhuis et al., 2011; Kim et al., 2012, Kim et al., 2016), and can cause disease as severe as that caused by “hypervirulent” *C. difficile* RT027 (Goorhuis et al., 2011). However, to date, no reports are available to evaluate the virulence characteristics of ST37 strains *in vitro* and in animal models of CDI in comparison with RT027 strains.

Here, we report a hypervirulent ST37/A-B+ *C. difficile* strain (Xy06) isolated from an ICU-hospitalized patient in China, where it caused severe infection symptoms. Xy07 is also an epidemic strain ST54 (A+B+) in our hospital and is included in this study as a clinical control strain from the same geographic region. We performed comparative genomic analyses of Xy06 with sequenced A-B+ *C. difficile* strains including two ST81 strains, and we further compared Xy06 with R20291, CD630E, and Xy07 in major virulence and pathogenicity *in vitro* and *in vivo*.

## 2 Materials and methods

### 2.1 Isolation and characterization of *C. difficile* strains

*C. difficile* Xy06 and Xy07 strains were isolated from stool samples of ICU hospitalized patients in China. *C. difficile* strains were routinely grown under anaerobic conditions in a Bactron III-2 anaerobic chamber in 3.7% Brain Heart Infusion (BHI) (Cat#53286, Sigma) supplemented with 0.05% yeast extract and 0.01% L-cysteine (BHIS) broth or on BHIS agar plates. Multi-locus sequence typing (MLST) of *C. difficile* strains was performed using a previously established method (Griffiths et al., 2010). Capillary PCR ribotyping was performed on Xy06 and Xy07 against the database of the Dutch National Reference Laboratory, a center recognized by the Europe Center for Disease Prevention and Control for the typing of *C. difficile* (Fawley et al., 2015),

### 2.2 Whole genome sequencing

Whole genome sequences of Xy06 and Xy07 were obtained using paired-end libraries and the sequencing-by-synthesis Illumina Hiseq 3000 platform. Approximately 200 Mb of clean data for each strain was obtained. Reads were assembled using Velvet (Zerbino and Birney, 2008), and contigs of 500 bp were scaffolded with SSPACE (Boetzer et al., 2011). This Whole Genome Shotgun project for Xy06 and Xy07 has been deposited at DDBJ/ENA/GenBank under the accession numbers JANFNF000000000 and JANFVQ000000000, respectively.

### 2.3 Phylogenetic analysis of A-B+ *C. difficile* strains

A literature search of Pubmed and Google Scholar databases with the search terms “toxin A-B+ *C. difficile* strain” was performed on August 6^th^, 2021, to identify previously isolated and sequenced A-B+ *C. difficile* strains. The appropriate genome sequence or accession number for each strain was sourced from GenBank (https://www.ncbi.nlm.nih.gov/genbank/). Additional information on the characteristics of the genomes was also gathered from the GenBank entries. To produce a phylogenetic tree, the sequence files or accession numbers were uploaded to the Type Strain Genome Server (TYGS) (Meier-Kolthoff and Göker, 2019). TYGS compared the genomes using the Genome BLAST Distance Phylogeny approach (GBDP) (Wu and Wu, 2013) and generated a minimum evolution tree. The tree data file was visualized using an online tool (ETE Toolkit Phylogenetic tree (newick) viewer).

### 2.4 Prophage analysis

Putative prophages were detected in the bacterial genome using PHASTER (upgrade 6) (Arndt et al., 2016), which screens for regions with high similarity to a database of phage genes. The software assigned prophage regions a score that determines whether they are considered complete (>90), questionable (70-90), or incomplete (<70). The sequences of predicted complete Xy06 prophages were aligned with sequences of previously isolated phages using ClustalW in MegaX software (version 11.0.13) on default settings. A Maximum-likelihood phylogenetic analysis was performed using default parameters and 100 bootstrap replicates. PaLoc gene sequences for *tcdA*, *tcdB*, *tcdR*, *tcdE*, and *tcdC* were downloaded from the GenBank entry for the reference strain CD630 (AM180355.1), and CdtLoc gene sequences for *cdtR*, *cdtA*, and *cdtB* were downloaded from the GenBank entry for the reference strain R20291 (FN545816.1). PaLoc and CdtLoc sequences were aligned against the putative prophage sequences using the online BLAST+ function of the Galaxy Web server (https://test.galaxyproject.org/).

### 2.5 Genome comparison

Blast Ring Image Generator (BRIG) (Alikhan et al., 2011) was used to compare non-toxigenic strains and the reference strain CD630. The upper, middle, and minimum identity percentage thresholds were set to 100%, 80%, and 50%, respectively. To identify transposons in Xy06, transposon sequences were mined from GenBank files of various strains using the boundaries of each element as previously reported (Brouwer et al., 2011). BLASTn comparisons between transposon sequences and the Xy06 genome were performed online through the National Center for Biotechnology Information (NCBI, https://blast.ncbi.nlm.nih.gov/Blast.cgi). The locations of alignments between the transposon sequences and the Xy06 genome were annotated on a GenBank file of the Xy06 genome so that BRIG software could illustrate the putative transposon-like regions.

### 2.6 Analysis of toxin loci

*C. difficile* genomes were downloaded from GenBank, and then the previously annotated pathogenicity locus (PaLoc) and surrounding genes of each strain were color-coded in Artemis (Rutherford et al., 2000). Upstream and downstream genes from PaLoc were identified in the genome annotations based on previous work describing the conserved *cdu* and *cdd* genes in these areas (Braun et al., 1996b). Color-coded annotations were imported into EasyFig software (version 2.2.5) (Sullivan et al., 2011) to generate BLAST comparisons. The binary toxin locus (CdtLoc) was illustrated in the same manner.

To generate alignments of PaLoc genes, the sequences of PaLoc genes were mined from the genome using Artemis and aligned using an online T-COFFEE aligner (Di Tommaso et al., 2011) or the online MPI Bioinformatics Toolkit T-COFFEE server (Gabler et al., 2020). The alignment of the CdtLoc of CDT-negative (CDT-) strains was performed by mining these sequences from the appropriate GenBank files before aligning the sequences using MAFFT (Katoh and Standley, 2013) on the MPI Bioinformatics Toolkit server (Gabler et al., 2020).

A phylogenetic tree of TcdB amino acid sequences was generated based on previous work (Shen et al., 2020a) modifications. TcdB amino acid sequences were mined from GenBank (see **Supplementary Material** for details) and aligned using Muscle Algorithm in MegaX software (Kumar et al., 2018). A Neighbor-joining tree was then constructed in MegaX with 1000 bootstrap replicates.

### 2.7 Toxin A and toxin B production determined by ELISA

Toxin production was measured at four different time points (12, 24, 48 and 72 hours post-inoculation). Single colonies of the strains were initially cultured in BHIS medium for 18-24 hours, and cultures (0.5% v/v) were inoculated into fresh TY medium (3% w/v tryptose, 2% w/v yeast extract, 0.1% w/v thioglycollate, pH 7.4), different strain cultures at each time-point were adjusted to the same OD600 value after incubation. Cultures at each time point were centrifuged at 12,000 × g for 10 min at 4°C. Each supernatant was filtered through a 0.45-μm membrane filter. Toxin production was measured by ELISA. 96-well plates were coated with 50 μl per well of anti-TcdA (Novus Biologicals, USA) or anti-TcdB (A1, Gene Tex, USA) antibody at a concentration of 0.5 μg/ml and plates were kept at 4°C overnight. The coated plates were washed with PBST (washing buffer, 1×PBS+0.05% Tween 20) and blocked with 60 μl per well of blocking buffer (PBS+5% dry milk) for 2 hours. After being washed with PBST, the plates were incubated with 50 μl of supernatants/well collected at 12, 24, 48, and 72 hours post-inoculation in TY medium at room temperature for 1.5 hours. After being washed with PBST, the plates were further incubated with 50 μl of HRP-Chicken anti-Tcd A antibody (1: 5000 dilution, Gallus Immunotech, USA) or 50 μl HRP-Chicken anti-TcdB antibody (1: 2500 dilution, Gallus Immunotech, USA) per well at 37°C for 1 hour. The plate was washed again, and each well was supplemented with 50 μl TMB substrate and was incubated for 20-30 minutes. 50 μl of stop solution (2 N of H2SO4) was added to each well and mixed well. The optical density of each well was determined immediately using a spectrometer (Biotek Synergy HT Multi-Mode Microplate Reader).

### 2.8 Cytotoxicity assay

The murine CT26 cells cultured in RPMI 1640 (Gibco) with 10% (vol/vol) FBS (Gibco), 10 mM L-glutamine (Gibco), and 100 μg/mL penicillin/streptomycin (Gibco) were seeded in 96-well plates and were cultured to more than 80% confluence followed by addition of 16 μl of *C. difficile* culture supernatants collected at 72 hours post-inoculation in TY medium. Cell rounding was visualized by phase-contrast microscopy every two hours. Approximately 100 cells from 5 random fields were counted, and the percentage of rounded cells was determined. The experiments were repeated twice, and triplicate wells were assessed for cell rounding in each experiment.

### 2.9 *C. difficile* spore preparation

*C. difficile* culture was streaked on pre-reduced BHIS agar medium and incubated under anaerobic conditions for 48 hours. One colony was scraped up and suspended in 5 ml of Columbia Broth (CB) in a sterile 15 ml plastic tube and incubated for 48 hours. 50 μl of culture was then inoculated into 20 ml CB in a 50 ml tube and incubated for 24 hours. The entire 20 ml culture was inoculated into 500 ml of Clospore medium (Special Peptone Mix (SPM; Oxoid; LP0072), 10 g/L; Yeast extract, 10 g/L; (NH4)_2_SO_4_, 0.6 g/L; MgSO_4_.7H_2_O, 0.12 g/L; CaCl_2_.2H_2_O, 0.08 g/L; K_2_CO_3_, 3.48 g/L; KH_2_PO_4_, 2.6 g/L; pH 7.9± 0.1) in a 1 L bottle for 7-10 days of incubation. The spore suspensions were centrifuged at 10,000×g for 10 minutes, and the pellets were washed five times each with sterilized H_2_O. The final pellet was resuspended in 10 ml of sterilized H_2_O for stock. The suspension was then layered on top of a 10 ml bed volume of 50% (wt/vol) sucrose in water. The gradient was centrifuged at 3,200 × g for 20 min. During the centrifugation, vegetative cells and debris were collected at the interface while spores migrated through the 50% sucrose bed as pellets. After centrifugation, the spore pellet was washed five times to remove the sucrose and suspended in water. All spore preparations were >99% free of vegetative cells and debris, and all spores appeared phase bright. Before the adherence assay, spore suspensions were heated at 60°C for 20 minutes to activate the remaining vegetative cells. Spore concentration was determined as previously described (Sorg and Dineen, 2009).

### 2.10 Measurement of sporulation rate

*C. difficile* strains were cultured to mid-log phase in BHIS medium supplemented with 0.1% taurocholate (Sigma) and 0.5% fructose at 37 °C in an anaerobic chamber. A mixture of 70:30 sporulation medium (70% SMC medium and 30% BHIS medium including 63 g Bacto peptone, 3.5 g protease peptone, 11.1 g BHI medium, 1.5 g yeast extract, 1.06 g Tris base, 0.7 g NH_4_SO4) was prepared. Cultures were subsequently diluted to an optical density at OD_600_ of 0.05 in 70:30 medium. Approximately 24 hours after the start of the stationary phase (T24), 1-2 ml of samples were taken from the cultures.

To measure ethanol-resistant spore formation, 500 µl of samples from the sporulation medium were removed from the anaerobic chamber and mixed 1:1 with 95% ethanol for 15 min to kill vegetative cells. Samples were then returned to the anaerobic chamber. 100 µl of ethanol-treated cultures were mixed with 100 µl of 10% taurocholate and were plated onto BHIS agar to induce germination of *C. difficile* spores. Ethanol-resistant CFU/ml was determined after incubation for 24 h. Ethanol-resistant CFU/ml was divided by the total CFU/ml of the non-ethanol-treated cultures. A minimum of three biological replicates were performed for each strain.

### 2.11 Determination of spore germination

The initiation of spore germination was monitored aerobically at OD_600_. C*. difficile* spore germination was initiated by suspending spores in the germination solution [(10 mM Tris (pH 7.5), 150 mM NaCl, 100 mM glycine, and 10 mM taurocholic acid)]. Spores were heat-shocked at 65°C for 30 minutes and then placed on ice. Next, 5 µl of spores were diluted into 995 µl of buffer with or without germinant and mixed, and the change in optical density at 600nm (OD_600_) was monitored. The ratio of the OD_600_ at time X (Tx) to the OD_600_ at time zero (T_0_) was plotted against time. Germination rates were determined using the slopes of the linear portions of the germination plots. Data are reported as the averages from three independent experiments with standard deviations.

### 2.12 Determination of the spore colony-forming efficiency

Spore suspensions were serially diluted and plated on BHIS agar supplemented with 0.1%(w/v) taurocholic acid (TA). Plates were incubated anaerobically at 37°C for 24 h and colony counts of the starting suspension were determined.

The number of phase-bright spores/ml of starting suspension was counted directly by phase-contrast microscopy using a hemocytometer counting chamber (Bright-Line). The germination efficiency was defined as the percentage of total spores that gave rise to colonies on BHIS agar with 0.1% (w/v) TA, calculated by c.f.u. per ml colony count/c.f.u. per ml direct count by microscopy × 100%.

### 2.13 Ability of *C. difficile* spores to adhere to HCT-8 cells

Human gut epithelial HCT-8 cells were cultured in RPMI 1640 (Gibco) with 10% (vol/vol) FBS, 10 mM L-glutamine, and 100 μg/mL penicillin/streptomycin. Following five days of incubation, HCT-8 cells (5 ×10^5^ cells per ml in a 6-well plate) were co-cultured with 100 μl of 5×10^7^ spores/ml in sterile deionized water and incubated for 100 minutes at 37°C in the anaerobic chamber. After incubation, the cell-spore mixture was washed with 1× PBS via centrifugation at 800× g for 1 minute to remove any unattached spores. This washing step was performed three times, and the supernatants from each step were collected to enumerate any spores not adhering to the cells. The spores in the supernatant were enumerated on pre-reduced BHI plus 0.1% taurocholic acid (w/v). Controls included PBS incubated with spores and RPMI incubated with spores. All cell culture assays were performed in triplicates. The percentage of spore adherence was calculated by using the following formula (initial CFU/ml - eluted CFU/ml)/initial CFU/ml.

### 2.14 Evaluation of *C. difficile* strain pathogenicity in the mouse model of CDI

Six-week-old C57BL/6 female mice were purchased from Charles River Laboratories, MA. All animal experiments were approved by the institutional committee for animal care and performed at the University of South Florida. Forty mice were divided into four groups. Group 1 (n=10) was challenged with spores of *C. difficile* R20291. Group 2 (n=10) was challenged with spores of *C. difficile* CD630E, and Group 3 (n=10) was challenged with spores of *C. difficile* Xy06. Group 4 (n=10) was challenged with spores of *C. difficile* Xy07. All mice were given drinking water containing a mixture of five antibiotics including kanamycin (0.4 mg/ml), gentamycin (0.035 mg/ml), colistin (0.042 mg/ml), metronidazole (0.215 mg/ml) and vancomycin (0.045 mg/ml) for five days, and then received autoclaved water for two days, followed by a single dose of clindamycin (10 mg/kg) intraperitoneal injection before challenge day (day 0). On the challenge day (day 0), mice in each group were challenged with 10^6^ C*. difficile* spores by gavage. Mice were monitored daily for a week after challenge with *C. difficile* spores for weight changes, survival, diarrhea, and other disease symptoms. Diarrhea was defined as wet tails along with loose or watery feces. The percentage of diarrhea was defined as the number of mice that developed diarrhea divided by the total number of mice in each group. The number of recorded dead mice included those that died after infection and those that were euthanized if weight loss was greater than 20%.

### 2.15 Statistical analysis

Animal survival was analyzed by Kaplan–Meier survival analysis with a log-rank test of significance. When comparing results for two groups, a Student’s unpaired *t*-test was used to assess significance. When comparing more than two groups, one-way analysis of variance (ANOVA) with *post hoc* analysis by Bonferroni tests was used. The results are expressed as the means ± standard errors of the means. Differences were considered significant if *p* < 0.05 (*). All statistical analyses were performed using GraphPad Prism.

## 3 Results

### 3.1 Ribotype and genomic characteristics of strain Xy06

To place the Xy06 strain in a phylogenetic context, we performed capillary PCR ribotyping and whole genome sequencing. Xy06 was determined to be an RT017 strain by capillary PCR ribotyping. Analysis of the draft genome sequence of Xy06 using a 7-gene multi-locus sequencing scheme confirmed the strain to be ST37, similar to other RT017 isolates. To analyze the Xy06 genome, we compiled sequenced A-B+ *C. difficile* strains from the literature for comparison (**Supplementary Table 1**). Strain Xy06 contains a 4,194,266 bp genome similar in size to previously reported A-B+ *C. difficile* genomes (**Supplementary Table 1**). The Xy06 genome has a GC content of 28.5%, in line with the 28-30% GC content typically observed in *C. difficile* genomes. The NCBI Prokaryotic Genome Annotation Pipeline (PGAP) predicted 48 tRNAs and 11 rRNAs.

### 3.2 Genomic comparison between Xy06 and the sequenced A-B+ *C. difficile* strains as well as the reference strain CD630 with a focus on PaLoc region

**Figure 1:**
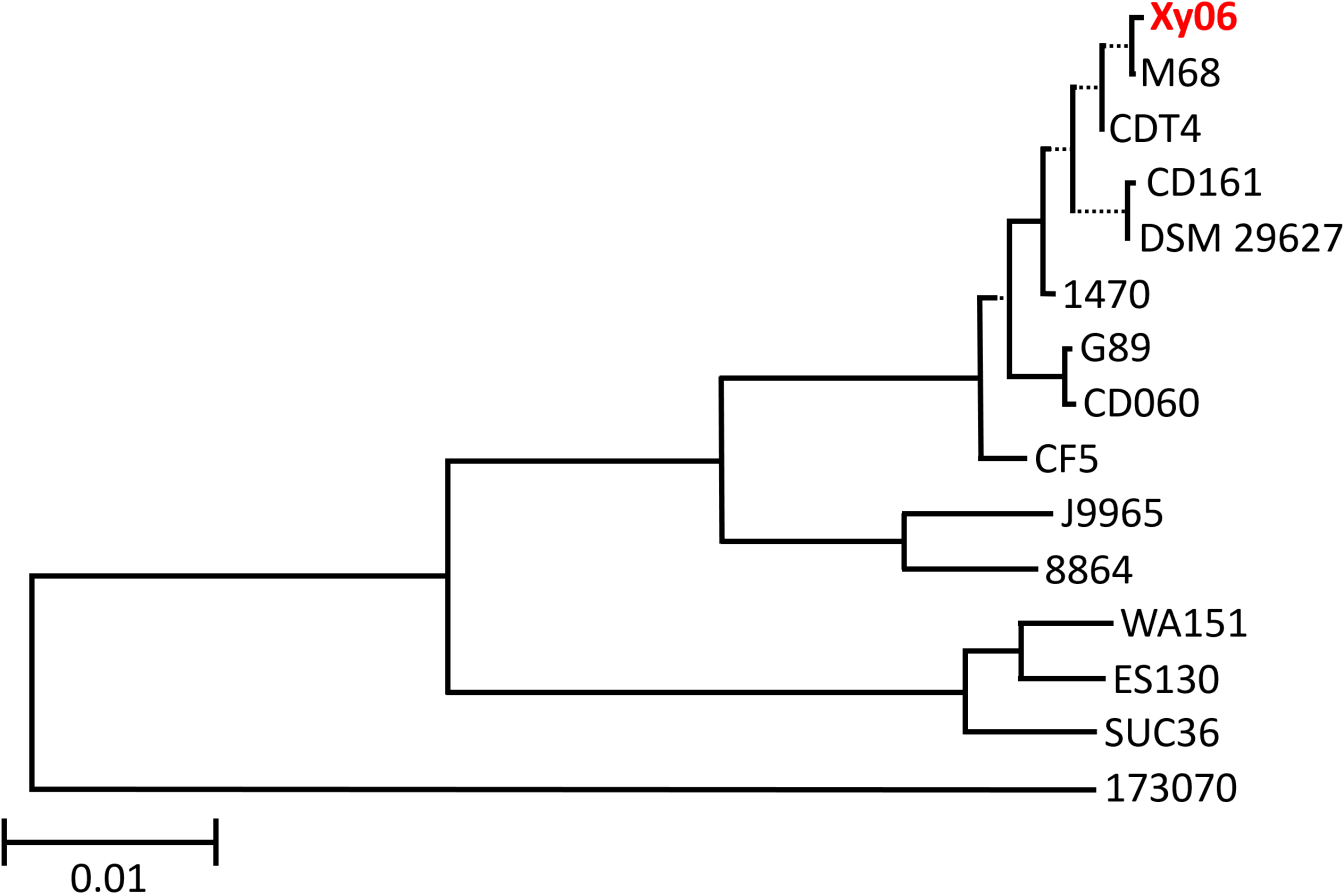
Phylogenetic tree of A-B+ *C. difficile* strains. A minimum evolution phylogenetic tree was generated using the Type (Strain) Genome Server. Xy06 is indicated in red. The scale bar indicates 0.01 nucleotide substitutions per site. Dotted lines separate select branches for easier viewing and do not indicate evolutionary distance.

Genomes of all A-B+ *C. difficile* strains in **Supplementary Table 1** were uploaded to the Type Strain Genome Server (TYGS) (Meier-Kolthoff and Göker, 2019) to generate a minimum evolution phylogenetic tree (**Figure 1**). The results indicate that Xy06 is most closely related to strains CDT4, DSM29627, M68, and CD161. Xy06, CD161, and CDT4 were all isolated from China, while DSM29627 and M68 were isolated in France and Ireland. Although not all the included strains have been ribotyped, Xy06 is more closely related to fellow RT017 strains (1470, M68, DSM 29627, CF5) than non-RT017 strains (88.64, SUC36, ES130, WA151, 173070). G89 and CD060, which are two ST81 strains, cluster with the ST37 strains used in this analysis (Xy06, M68, CDT4, CD161, DSM 29627, 1470, and CF5). A few prior studies have established that ST81 and ST37 strains are closely related (Wang et al., 2018b; Su et al., 2021a), which is reflected in our analysis.

In general, the pathogenicity locus (PaLoc) of *C. difficile* comprises a 19.6 kb region encoding five genes, including *tcdA* and *tcdB*. However, variant PaLocs have been noted in a minority of strains (Monot et al., 2015). Other genes in the locus include *tcdR*, *tcdC*, and *tcdE.* Specifically, *tcdR* is involved in the positive regulation of toxin transcription, *tcdC* may negatively regulate toxin transcription, and *tcdE* may facilitate toxin secretion (Braun et al., 1996a; Monot et al., 2015).

**Figure 2:**
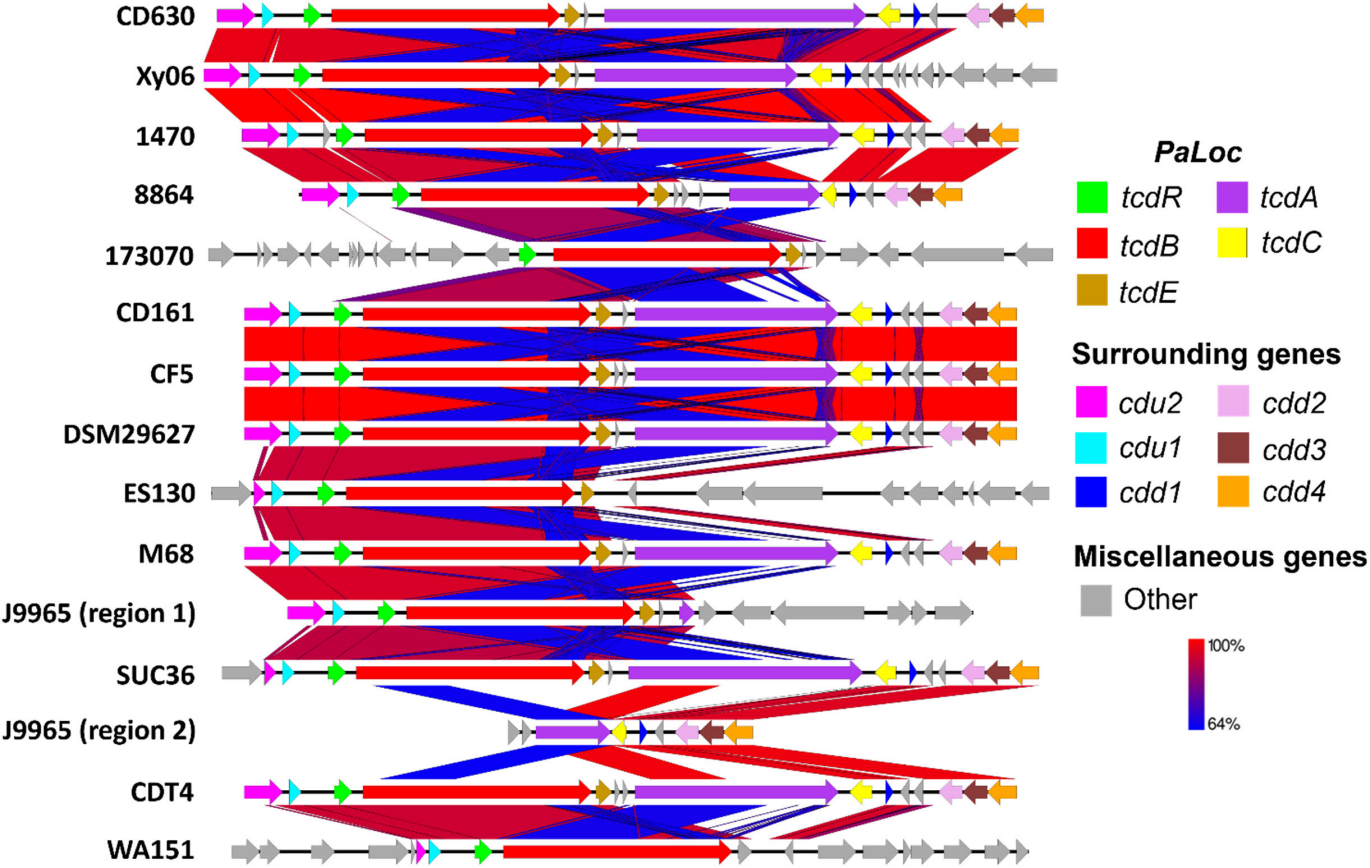
Comparative sequence analysis of PaLoc region. *C. difficile* typically encodes the genes *cdu2* (pink) and *cdu1* (light blue) upstream of PaLoc and *cdd2* (pink), *cdd3* (maroon), and *cdd4* (orange) downstream of PaLoc, so these were used as boundaries for visualizing the genome below. PaLoc-adjacent genes were visualized for strains lacking the above surrounding genes (e.g., 173070). A-B+ *C. difficile* strains display an atypical organization of their PaLoc region and surrounding genes, such as deletions in *tcdA* (e.g., Xy06) or multiple genes (e.g., 173070, ES130, J9965). Blue BLAST alignments indicate regions with relatively lower (64%) shared identity, while red regions indicate high similarity.

We examined how the PaLoc region and surrounding genes of strain Xy06 compared to other A-B+ *C. difficile* strains and the A+B+ reference strain CD630 (**Figure 2**). Upstream of the PaLoc region, *C. difficile* genomes typically encode *cdu1* and *cdu2*, while downstream genes include *cdd2*, *cdd3*, and *cdd4* (Braun et al., 1996b). Xy06 is an exception to this trend, as the *cdd2-4* genes are not encoded directly downstream of the PaLoc region, but rather are ~3Mbp upstream of the other PaLoc genes. A handful of other A-B+ strains (173070, ES130, and WA151) are similar in that the usual downstream genes are not adjacent to the PaLoc region. On another note, the upstream *cdu2* and *cdu1* genes, while intact in strain Xy06, are noted to be either absent in strain 173070 or show a deletion in *cdu2* in strains ES130, SUC36, and WA151.

#### 3.2.1 Variations in *tcdA*

The *tcdA* gene of Xy06, like the *tcdA* genes of the other A-B+ strains, is visually shorter than that of CD630 (**Figure 2**). First, we aligned the *tcdA* sequence of Xy06 and other strains using the T-coffee algorithm on an online server (Di Tommaso et al., 2011). Second, we determined the length of each *tcdA* gene using the online ENDMEMO DNA/RNA Length Calculator tool (**Table 1**). It was determined the Xy06 *tcdA* exhibits a ~1.8 kB deletion relative to the wild-type CD630 *tcdA*. Strains 1470, CD161, CDT4, CF5, DSM29627, and M68 have deletions of virtually identical sizes. Upon translating the DNA sequence of the above strains’ *tcdA* gene, we detected a premature stop codon at AA position 47. These findings align with a prior study that reported these mutations in strain 1470 (Von Eichel-Streiber et al., 1999).

**Table 1:** Size of PaLoc genes in reference strain and A-B+ *C. difficile* strains.

Strain	*tcdA*		*tcdB*		*tcdR*		*tcdE*		*tcdC*		*tcdE-tcdA*	
	Length	Change	Length	Change	Length	Change	Length	Change	Length	Change	Length	Change
CD630 (A+B+ reference)	8133	–	7101	–	555	–	501	–	699	–	727	–
Xy06	6309	-1824	7104	+3	555	0	501	0	699	0	718	-9
1470	6310	-1823	7104	+3	555	0	501	0	699	0	718	-9
8864	2840	-5293	7104	+3	555	0	499	-2	465	-234	1840	+1113
173070	–	–	7101	0	555	0	500	-1	–	–	–	–
CD161	6310	-1823	7104	+3	555	0	501	0	699	0	718	-9
CDT4	6310	-1823	7104	+3	555	0	501	0	699	0	718	-9
CF5	6310	-1823	7104	+3	555	0	501	0	699	0	718	-9
DSM29627	6310	-1823	7104	+3	555	0	501	0	699	0	718	-9
ES130	–	–	7104	+3	555	0	432	-69	–	–	–	–
J9965	501 + 2342	-5290	7104	+3	555	0	502	+1	465	-234	727	0
M68	6310	-1823	7104	+3	555	0	501	0	699	0	719	-8
G89	6229	-1904	7104	+3	555	0	501	0	699	0	719	-8
CD060	6310	-1823	7104	+3	555	0	501	0	699	0	720	-9
SUC36	7263	-870	7101	0	555	0	501	0	660	-39	727	0
WA151	–	–	7104	+3	555	0	–	–	–	–	–	–

Strain 8864 was found to have a ~5.3 kB deletion in *tcdA*, which is somewhat smaller than the previously reported figure of ~5.9 kB (Soehn et al., 1998). Translating the gene also indicated the presence of a stop codon at AA 699 before the end of the polypeptide sequence. It has also been noted that 8864 encodes a ~1kb insertion between *tcdE* and *tcdA* (Soehn et al., 1998). Our calculations in **Table 1** confirm this result and demonstrate that no other A-B+ strain in our analysis has this feature. SUC36 displays the smallest deletion (<1 Kb) of the analyzed strains. Finally, strains 173070, ES130, and WA151 exhibit deletions of the entire *tcdA* and *tcdC* genes.

Another unique A-B+ strain is J9965. While Xy06 and the other strains contain a single PaLoc region, J9965 exhibits a split PaLoc. The first study to report on the J9965 (Rupnik et al., 2003) classified the strain in toxinotype XVII, and it appears that their PCR experiment successfully amplified the first, smaller tcdA fragment (J9965 region 1, **Figure 2**). The second, larger fragment that we report (J9965 region 2, **Figure 2**) was not detected by the prior study. The researchers noted that their amplified *tcdA* gene was ~1.9kb smaller than the *tcdA* of 8864, which is similar to the difference between the 8864 *tcdA* and J9965 *tcdA* fragment 1 lengths we calculated using sequencing data (~2.3kB, **Table 1**). Regarding the formation of the J9965 *tcdA* fragment 2, it is possible that a past recombination event split the PaLoc region at the *tcdA* gene, thereby abolishing *tcdA* expression.

#### 3.2.2 Variations in *tcdB*

**Figure 3:**
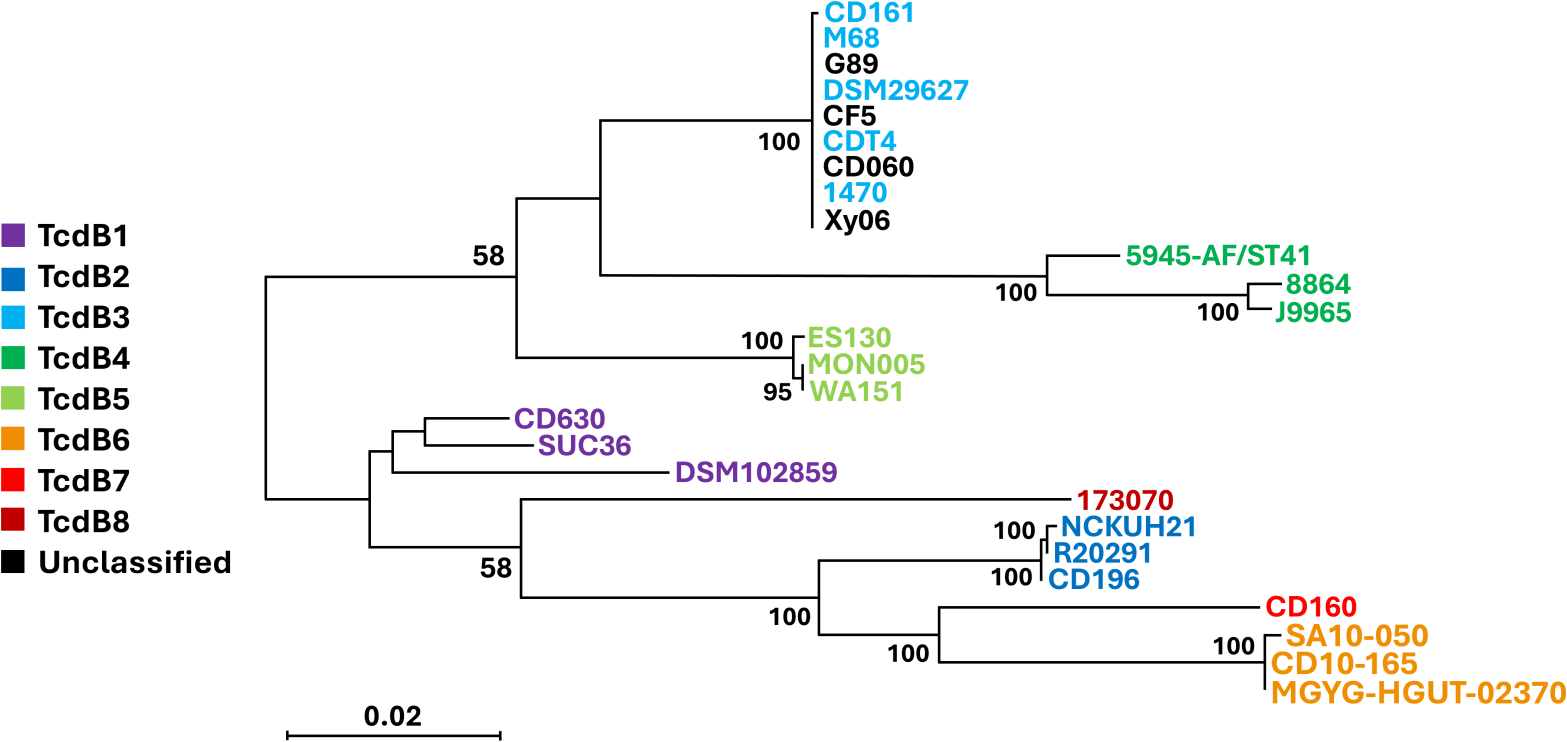
Phylogenetic tree of A-B+ *C. difficile T*cdB subtypes. A neighbor-joining phylogenetic tree of TcdB amino acid sequences from the A-B+ strains examined in this study and other strains representing the various subtypes of TcdB. Xy06 TcdB is clustered with several A-B+ strains that encode TcdB3 (light blue). The scale bar indicates 0.020 amino acid substitutions per site. Bootstrap values of 50 or greater are displayed.

TcdB production is essential for the virulence of *C. difficile*. All A-B+ strains except for those in toxinotype XVI (strain SUC36) have been reported to contain a hybrid *tcdB* that is homologous to *TcsL* of *C. sordellii* (Chaves-Olarte et al., 2003). In line with this observation, strain Xy06 displays 83.68% similarity over 99% coverage between its *tcdB* and the *tcsL* toxin gene. Based on the T-coffee alignment of the *tcdB* genes of A-B+ strains as well as the calculated lengths of the *tcdB* genes (**Table 1**), we confirmed that the *tcdB* gene has a consistent size across A-B+ strains. A previous study classified TcdB into eight subtypes (Shen et al., 2020b), while another study demonstrated that certain subtypes may be more damaging than others in disease models (Pan et al., 2021). To determine the TcdB subtype of Xy06 and how it compares with other A-B+ strains as well as the hypervirulent R20291 strain, a neighbor-joining phylogenetic tree of TcdB amino acid sequences was constructed with annotated TcdB subtypes based on a previous subtyping of *C. difficile* TcdB (**Figure 3**) (Shen et al., 2020b). Just as Xy06 *tcdA* was similar to the *tcdA* of strains 1470, CD161, CDT4, CF5, DSM29627, and M68, the TcdB sequences were also highly similar between these strains as evidenced by the cluster of TcdB3 strains and Xy06 on the phylogenetic tree. The results suggest that Xy06 encodes a TcdB3 subtype toxin B, distinct from the TcdB1 subtype of CD630 and the TcdB2 subtype of R20291. Interestingly, the two ST81 strains used in this analysis, G89 and CD060, also encode TcdB3 toxins that are identical to that encoded by Xy06. Studies so far suggest that ST81 strains’ increased presence in healthcare facilities is, in part, due to their increased sporulation, motility, and antibiotic resistance (Wang et al., 2018b; Su et al., 2021a). Certain ST81 strains may express less *tcdB* at the mRNA level (Wang et al., 2018b), but no major mutations have been noted in ST81 TcdB relative to ST37 TcdB (Wang et al., 2018b; Su et al., 2021a). Our analysis shows that the G89 and CD060 encode the same TcdB sequence as Xy06 and other ST37 strains in our dataset, further suggesting that differences in virulence between ST37 and ST81 A-B+ strains is due to factors other than TcdB.

#### 3.2.3 Variations in *tcdE*, *tcdR*, and *tcdC*

The T-coffee alignment (**Supplementary Figure 1**) showed highly similar *tcdE* sequences across the strains surveyed. One exception is ES130, which displays a 73 bp deletion at the 5’ end of the gene and a 4bp insertion at the 3’ end relative to the other A-B+ strains analyzed.

Finally, *tcdC* and *tcdR* were also generally well conserved among A-B+ strains. TcdR showed no significant deletions or additions in the strains analyzed (**Supplementary Figure 2**). However, some strains did display variations in *tcdC* (**Supplementary Figure 3**). J9965 and 8864 have identical 234 bp deletions in their *tcdC* genes, whereas SUC36 has a smaller 39 bp deletion (**Supplementary Figure 3**, **Table 1**).

### 3.3 Xy06 is a robust TcdB producer

**Figure 4:**
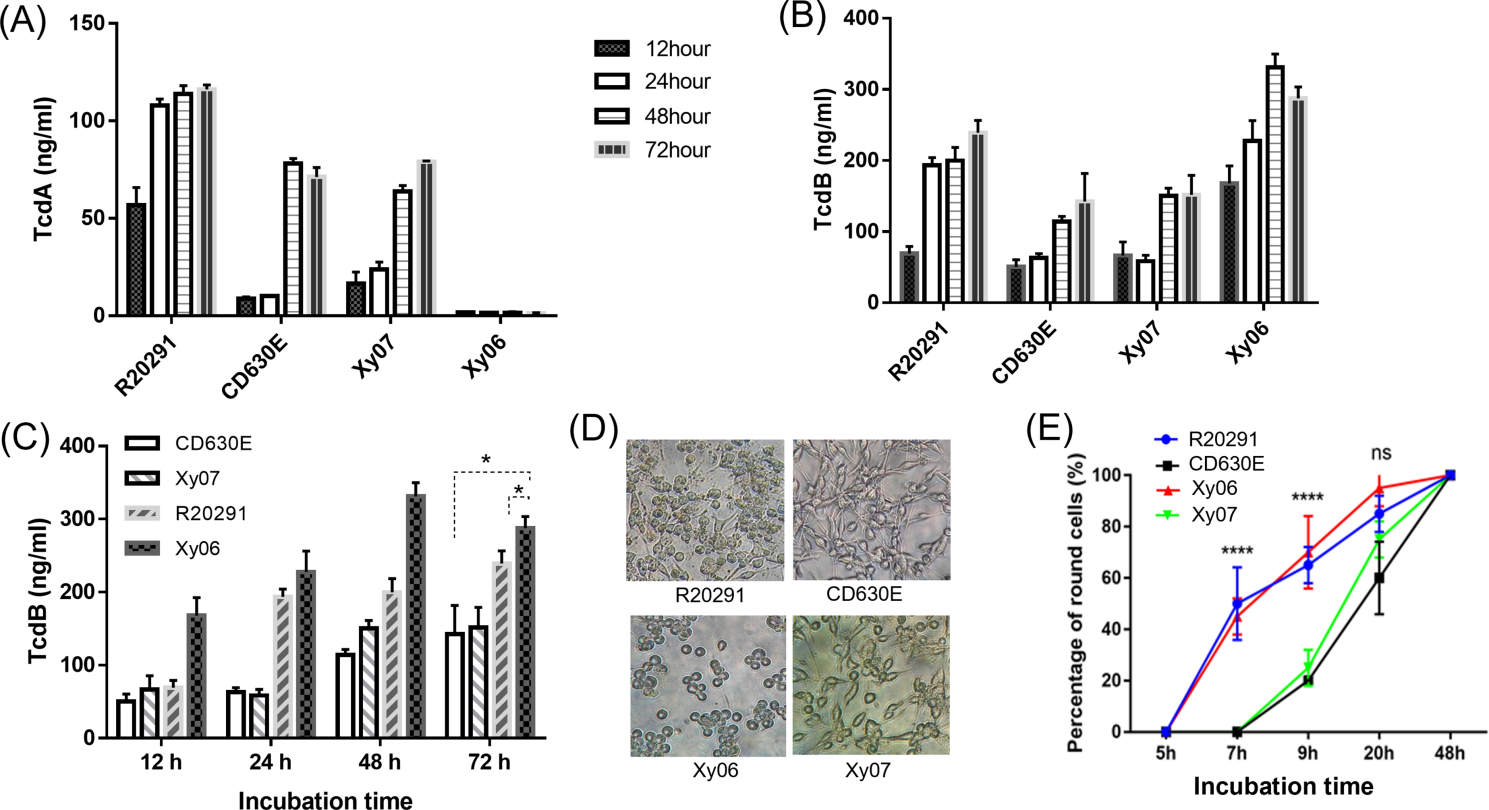
Toxin production in strains Xy06 and Xy07. TcdA **(A)** and TcdB **(B)** concentrations of *C. difficile* culture supernatants at different time points. **(C)** TcdB concentrations of *C. difficile* culture supernatants were compared at the same time point. *P < 0.05 (t-test). **(D)** Cytotoxicity of *C. difficile* culture supernatants. *C. difficile* culture supernatants (16 μl) collected at 72 hours post-inoculation in TY medium were added to CT26 cells seeded in 96-well plates. CT26 cell rounding was visualized by phase-contrast microscopy (40X) at 20 hours post-treatment. **(E)** Percentage of round cells at different time points (****P<0.0001, one-way ANOVA). Data are presented as mean ± SEM (n = triplicate). ns- not significant.

TcdA and TcdB production of Xy06 was evaluated in comparison with strains R20291 (ST1), CD630E (ST54), and the Xy07 strain that was isolated from a patient at the same institute as Xy06. Whole genome sequencing showed that Xy07 belongs to ST54. Consistent with this, Xy07 was determined to be an RT012 strain by capillary PCR ribotyping. Xy06 strain did not produce detectable TcdA by ELISA, while R20291, CD630E, and Xy07 produced both TcdA and TcdB. The highest concentrations of both toxins were detected at 48 h or 72 h post-inoculation. Of the strains tested, R20291 showed the highest level of TcdA production (> 100 ng/ml, **Figure 4A**). Of note, Xy06 showed the highest level of TcdB production (331 ± 18.50 ng/ml) at 48 hours post-inoculation (**Figures 4B, C**).

Even though Xy06 only produces TcdB, its culture supernatant induced comparable cell rounding to the culture supernatant from R20291 (A+B+) strain (**Figure 4D**), with significantly higher cell cytotoxicity compared with CD630E and Xy07 at 7 hours and 9 hours post-inoculation (one-way ANOVA, P<0.0001) (**Figure 4E**). Significant cell rounding was already evident at 7 hours post-inoculation for cells exposed to culture supernatants of Xy06 (ca. 45%) and R20291 (ca. 50%), whereas limited toxicity was observed for the two ST54 strains at this time point. Nearly complete cell rounding (95%) was visualized at 20 h post-inoculation with Xy06 culture supernatant. Thus, Xy06 culture supernatant induced a faster rounding of CT26 cells compared to CD630E and Xy07 (**Figure 4E**). Taken together, our data demonstrate that Xy06 is a robust TcdB producer capable of inducing cytopathic effects similar to RT027 strains.

### 3.4 Xy-06 shows strong binding to human gut epithelial cells

**Figure 5:**
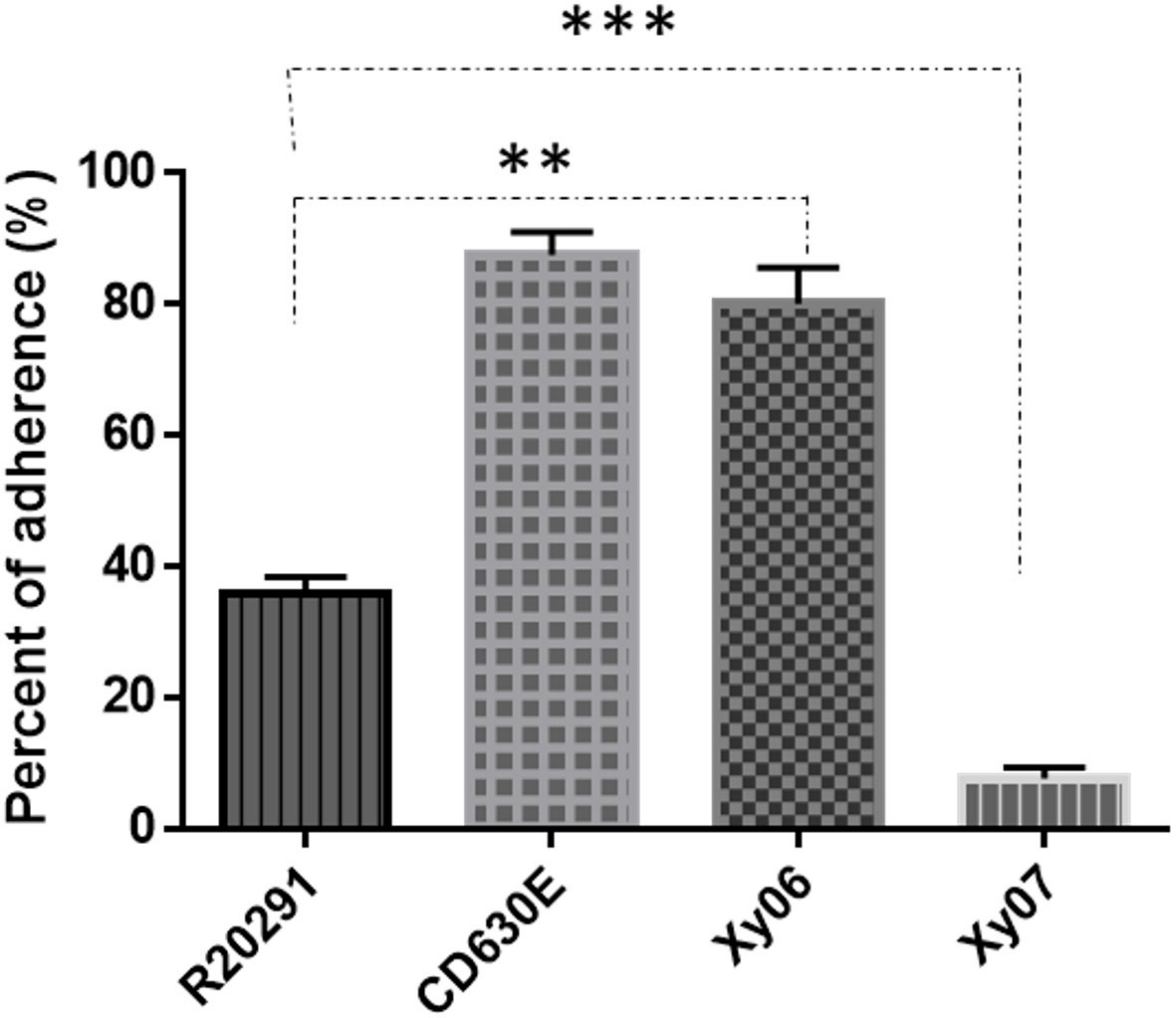
Adhesion of *C. difficile* spores to HCT-8 cells. HCT-8 cells were incubated with spores for 100 minutes under anaerobic conditions, and the relative number of adherent spores was calculated. Data are from three biological replicates and reported as mean ± SEM. **p=0.0097 (Xy06 VS R20291); p=0.8011 (Xy06 VS CD630E); *** p<0.0001 (R20291 VS Xy07).

The adhesion and persistence of *C. difficile* spores in the gut are the root cause of CDI and recurrence (Castro-Cordova et al., 2021). We also determined the binding capability of spores to HCT-8 human gut epithelial cells. Of the strains tested, Xy06 spores showed comparable adherence efficiency to CD630E spores (p=0.8011), and the adherence efficacy of Xy06 spores was significantly higher than R20291 spores (unpaired t-test, p=0.0097) (**Figure 5**).

### 3.5 Sporulation and germination capabilities of Xy06

**Figure 6:**
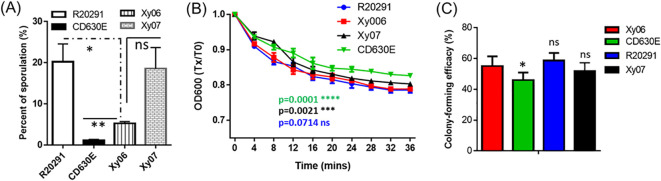
Sporulation and germination rates of *C. difficile* strains. **(A)** Sporulation. *P<0.05; **P<0.01. **(B)** Germination: Purified *C. difficile* spores were suspended in buffer supplemented with taurocholic acid and glycine to induce germination. Germination was monitored by plotting the ratio of the OD_600_ at a given time to the OD_600_ at time zero. Data are from three biological replicates and reported as mean ± SEM. ****p=0.0001(CD630E vs Xy06); ***p=0.0021 (Xy07 vs Xy06); ns: p=0.0714 (R20291 vs Xy06). **(C)** Spore colony-forming efficiency. Spore suspensions were serially diluted and plated on BHIS agar supplemented with 0.1%(w/v) taurocholic acid (TA). The spore colony-forming efficiency was defined as the percentage of total spores that gave rise to colonies on BHIS agar with 0.1% (w/v) TA, calculated by c.f.u. per ml colony count/c.f.u. per ml direct count by microscopy × 100%. Data are from three biological replicates and reported as mean ± SEM. * p=0.0276 (CD630E vs Xy06); ns: no significance (R20291, Xy07 vs Xy06). ns- not significant.

Sporulation and germination play critical roles in *C. difficile* pathogenesis and transmission. Sporulation and germination capabilities of Xy06 were also determined in comparison with strains R20291, CD630E, and Xy07. Xy06 is significantly more efficient in sporulation (p=0.0065) (**Figure 6A**) and germination (p=0.0001) (**Figure 6B**) than CD630E, significantly less efficient in sporulation than R20291 (P=0.0398) (**Figure 6A**) while comparable with R20291 in germination (P=0.0714). In addition, Xy06 is less efficient in sporulation (ns, p=0.0666) while significantly more efficient in germination than Xy07 (p=0.0021) (**Figure 6B**). To further assess the germination efficiency of *C. difficile* Xy06, Xy07, R20291 and CD630E, the colony-forming efficiency of spores was determined. Xy06 spores are significantly more efficient in forming colonies than CD630E spores but comparable with R20291 and Xy07 spores (**Figure 6C**). Our data indicate that Xy06 readily sporulates and germinates.

### 3.6 Xy06 induces strong symptoms in a mouse model of CDI

**Figure 7:**
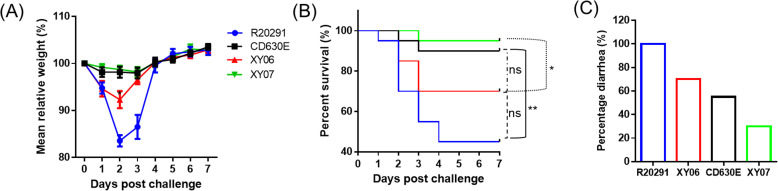
Virulence of Xy06 in the mouse model of CDI. Four groups of mice (n = 20) were challenged with 10^6^ spores of Xy06, Xy07, CD630E and R20291, respectively. Mice were monitored for one week for weight changes **(A)**, survival **(B)**, and diarrhea **(C)**. Animal survival was analyzed by Kaplan–Meier survival analysis with a log-rank test of significance. The mean relative weight of mice was analyzed for significance using a Student’s t-test. Xy06 challenged group lost significantly more weight at day 2 post-infection compared to CD630E challenged group (p=0.0145). R20291 challenged group lost significantly more weight at days 2 and 3 post-infection than Xy06 challenged group (p=0.0015 and p=0.0076, respectively) **(A)**. (*p < 0.05, **p < 0.01 and **p < 0.01). ns- not significant.

The pathogenicity of Xy06 *in vivo* was evaluated in the mouse model of CDI in comparison with strains R20291, CD630E, and Xy07. *C. difficile* R20291 challenged group showed a mortality rate of 55% (**Figure 7B**) and a diarrhea rate of 100% (**Figure 7C**). The Xy06 challenged group also presented a high death rate (30%) and a diarrhea rate (70%), which were higher than those of the CD630E challenged group ((10% death rate (p=0.1164 between Xy06 and CD630E groups) and 55% diarrhea rate, **Figure 7C**) and were also significantly higher than those of the Xy07 challenged mice ((5% death rate (p=0.0362 between Xy06 and Xy07 groups) and 30% diarrhea rate, **Figure 7C**). The Xy06-challenged group lost significantly more weight at post-infection day 2 compared to the CD630E-challenged group (**Figure 7A**). The R20291-challenged group lost significantly more weight at post-infection days 2 and 3 than the Xy06-challenged group (p=0.0015 and p=0.0076, respectively). Taken together, Xy-06 was more virulent than strain CD630E and Xy07, while less virulent with no significance than strain R20291 in mortality (p=0.1556 between Xy06 and R20291 groups) in the mouse model of CDI.

## 4 Discussion

Though initially identified in Asia, RT017/ST37 strains have also been identified in other countries such as Canada, Argentina, Australia, Israel, South Africa, and throughout Europe (Cairns et al., 2017). Strikingly, the proportion of RT017/ST37 is much higher in China than other areas of the world (Wu et al., 2022). Several publications reported that infections with the strains of type ST37/RT017 are the leading epidemic healthcare-associated CDIs in China (Yan et al., 2013; Jin et al., 2017; Yang et al., 2017), but factors contributing to their success as a pathogen in this region are still unknown. Although RT027 strains cause outbreaks in the Western world and are associated with increased morbidity and mortality, RT027 cases in China were only occasionally reported, and no significant outbreaks and death cases in China have been documented (Cheng et al., 2016; Jia et al., 2016; Wu et al., 2022). There are some indications that RT017 strains might exhibit clinical characteristics similar to hypervirulent RT 027 strains (Goorhuis et al., 2011).

Here, we provide experimental evidence supporting these indications. In particular, Xy06 showed a higher production of TcdB than CD630E and Xy07 (ST54) clinical strains under laboratory conditions. Notably, Xy06 produced more TcdB and at a faster rate than R20291 (highest TcdB production at 48 hours for Xy06 and at 72 hours for R20291 and other strains). In the cytotoxicity assay, Xy06 culture supernatant also induced a high level of cell cytotoxicity similar to R20291. At present, the reason for the high toxin expression is unknown. Regulation of toxin expression is complex. In some studies, mutations within the *tcdC* gene have been postulated to affect its function as a negative regulator (Matamouros et al., 2007), but others have not found such a relation (Cartman et al., 2012). In this study, we did not find any deletion or mutation of *tcdC* that might inactivate TcdC and lead to overexpression of *tcdA* and *tcdB*.

TcdA and TcdB share 49% identity and 63% similarity and share a similar domain structure (Pruitt and Lacy, 2012). The relative role of each toxin in the pathophysiology of CDI has long been a matter of debate, though TcdB is considered to be the more potent cytotoxin. The sequence of PaLoc comparison showed that the Xy06 genome carries a truncation of the *tcdA* gene and a functional tcdB gene, which codes for a hybrid TcdB (TcdB3) that is homologous to TcsL of *C. sordellii*. This hybrid *tcdB* gene seems highly conserved in RT017 *C. difficile* strains, and TcdB3 has been reported to target different sets of Rho GTPases in mammalian cells in comparison with TcdB from non-RT017 strains (Chaves-Olarte et al., 1999; Genth et al., 2014). However, a recent study showed that TcdB1 (from representative strain CD630), TcdB2 (from representative strain R20291), and TcdB3 exhibited comparable potency in a direct toxin challenge by intraperitoneal injection in mice (Shen et al., 2020b), though these TcdB variants induced different pathological effects and are highly diverse in their receptor preference (Pan et al., 2021). Even though Xy06 produces TcdB faster and in larger quantity than R20291 and Xy06 spores show stronger binding to HCT-8 cells than R20291 spores at least *in vitro*, it is less virulent, though not significant, than R20291 in the mouse model of CDI, indicating the complexed interplays among virulence factors and colonization, especially TcdA, TcdB and CDT *in vivo* that lead to the disease symptoms. In this regard, one of the limitations of this study is the missing data on *C. difficile* numbers and toxin levels in fecal samples of the infected mice, which may indicate the toxin production and *C. difficile* colonization *in vivo*.

Notably, our results showed that the Xy06 genome clusters closely with those of other RT017 strains (**Figure 1**), and the PaLoc genes (*tcdA, tcdB, tcdC, tcdR*, and *tcdE*) are highly conserved among all RT017 strains (**Figure 3** and **Supplementary Figures 1**, **Supplementary Figure 2**, **Supplementary Figure 3**). This suggests that the Xy06 virulence characteristics, such as the strong adherence to HCT-8 cells, ability to form spores and germinate, and robust toxin production may be representative of other RT017 strains.

*C. difficile* genomes can harbor multiple prophages (Heuler et al., 2021). Some *C. difficile* phages, such as phiCD119 and phiCD38-2, have been shown to regulate toxin expression, while the phage phiSemix9p1 encodes the entire binary toxin locus (Fortier, 2018). Given the potential that phages may influence *C. difficile* virulence, we searched for putative prophages in the Xy06 genome and prophage-encoded toxin genes. In Xy06, PHASTER predicted eight incomplete prophage-like regions and two putative complete prophages (**Supplementary Table 2**). The first complete prophage (sequence 2 in **Supplementary Table 2**) was predicted to have a length of 44.5 Kb and a GC content of 29.4%. Meanwhile, the second complete prophage (sequence 4 in **Supplementary Table 2**) was predicted to have a length of 50 Kb and a GC content of 27.8%. These statistics of both prophages are in agreement with the length and GC% ranges observed in other *C. difficile* bacteriophages (Heuler et al., 2021). We sought to determine if either of the complete prophages (Prophage 2, Prophage 4) were related to previously sequenced *C. difficile* phages implicated in virulence mechanisms. A Maximum-likelihood phylogenetic analysis shows that both Prophage 2 and Prophage 4 of strain Xy06 are most closely related to phage JD032. JD032 infection has been shown to downregulate a hemolysin, the sporulation-related sigma factor *sigH*, and two surface-proteins of *C. difficile* related to adhesion (Cwp2, Cwp66) (Li et al., 2020). The authors of this study hypothesized that the initiation of JD032 infection could attenuate the virulence of its host strain, which raises the possibility of a similar relationship between strain Xy06 and its prophages. Also, like JD032, the prophages of Xy06 do not appear to encode either PaLoc genes or CdtLoc genes based on BLAST analysis.

Several conjugative transposons (CTns) and transposable elements (Tns) have been identified in *C. difficile* strains, such as CD630 (Brouwer et al., 2011). CTns and Tns can significantly impact the virulence of strains that encode them. For example, the Tn*5398* element encoded in CD630 confers tetracycline and erythromycin resistance (Mullany et al., 1995). The strong similarity between transposons of various *C. difficile* strains suggests that bacteria frequently exchange these regions through horizontal gene transfer (Brouwer et al., 2011). To identify CTns and Tns in Xy06, transposon sequences were mined from GenBank files of various strains using the boundaries of each element as previously reported (Brouwer et al., 2011). These were aligned with the Xy06 genome using NCBI BLAST, and the results were reported in **Supplementary Table 3**. The sequences of CTn*5* and Tn*6106* produced strong alignments with regions of the Xy06 genome that are at least 90% the size of the reference transposon sequences and shared at least 98% identity. CTn5-like elements in *C. difficile* strains such as R20291 and QCD-23M63 encode putative sigma factors that could potentially impact the transcription profile of CTn5-encoding strains (Brouwer et al., 2011). Tn6106, unlike CTn5, has not been shown to be excisable, but it does encode putative recombinases and a putative sigma factor among other genes (Brouwer et al., 2011). Additional six elements (CTn*1*, CTn*2*, CTn*3*, Tn*6073*, Tn*6107*, and Tn*6110*) aligned with sequences of the Xy06 genome also showed high shared identity (at least 85%) but were shorter than the reference transposon sequence (50-69% query coverage). Four transposons (CTn*6*, Tn*5398*, Tn*6103*, and Tn*6104*) displayed poor quality alignments because of low query cover (below 50%). CTn*4* and Tn*6105* did not produce any significant alignment according to the BLAST algorithm. It will be interesting to investigate the exact roles of these CTn and Tns in Xy06 physiology and fitness in the future.

In summary, Xy06 is more virulent than strains CD630E and Xy07, and is comparable to strain R20291 in virulence. Xy06 carries two putative prophages and several mobilizable elements, which might contribute to its increased virulence and fitness.

## Data Availability

Data will be available from the corresponding authors once requested. The genomes of *C. difficile* strains Xy06 and Xy07 presented in this study were deposited in the GenBank repository (https://www.ncbi.nlm.nih.gov/genbank/), accession numbers JANFNF000000000 and JANFVQ000000000, respectively.
