# Supplementary material for: Genomic and phenotypic characterization of a Clostridioides difficile strain of the epidemic ST37 type from China
Source: Front Cell Infect Microbiol. 2024 Oct 18;14:1412408. doi: 10.3389/fcimb.2024.1412408 (PMC11527712; doi:10.3389/fcimb.2024.1412408)
Supplement: Supplementary file 1 [file DataSheet1.docx]

Supplementary Material

# Supplementary Figures


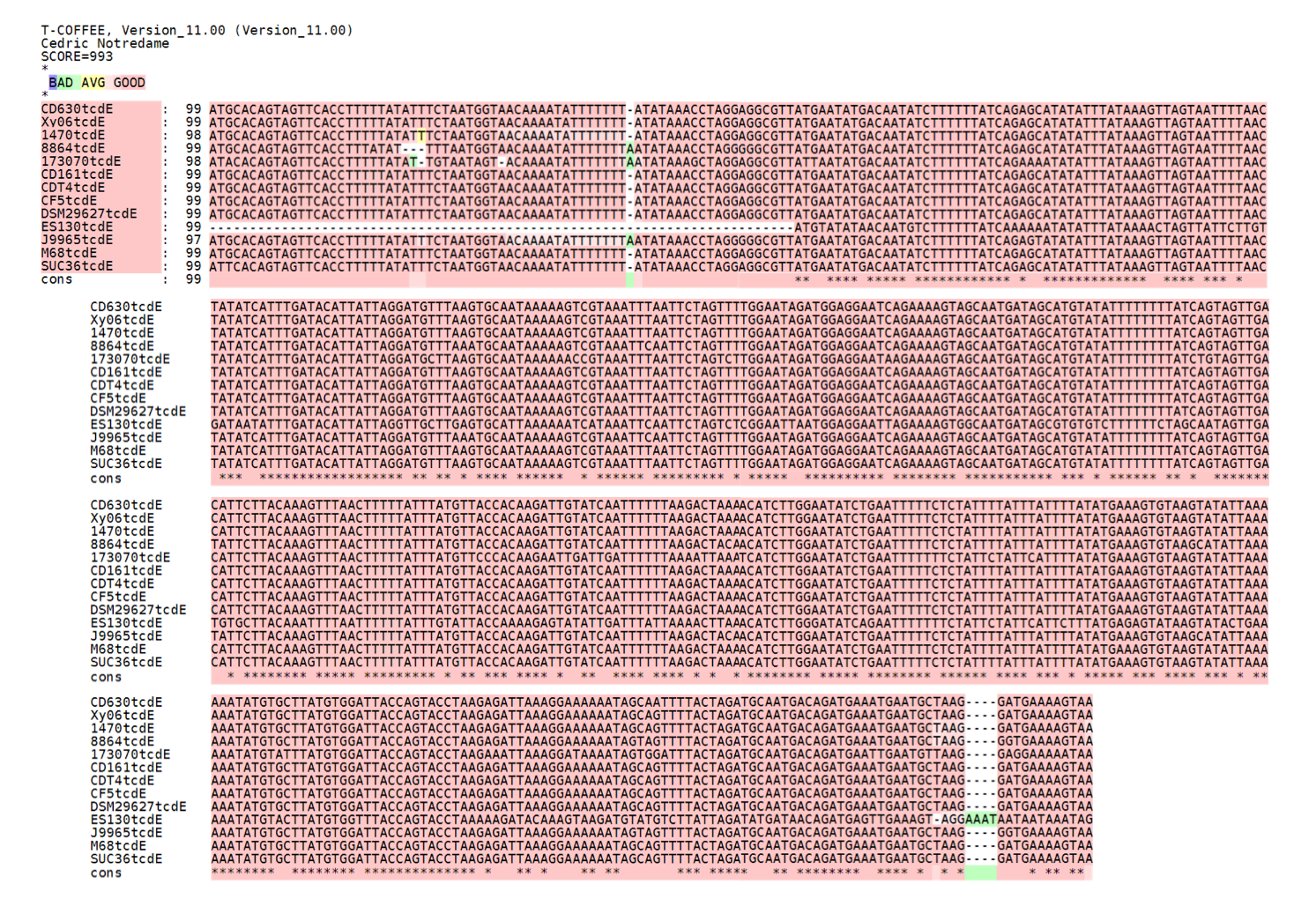
**Supplementary Figure 1.** Toxin A-B+ *tcdE* alignment. T-coffee alignment of *tcdE* nucleotide sequences. Dashes indicate gaps in the alignment, and asterisks (*) in the bottom row indicate conserved (cons) nucleotide sequence. The alignment score is 993 out of 1000 for the total similarity of all sequences, and the comparison of individual sequences to the group is scored out of 100. The highlighted color indicates whether the alignment is of bad (blue), average (yellow), or red (good) quality.


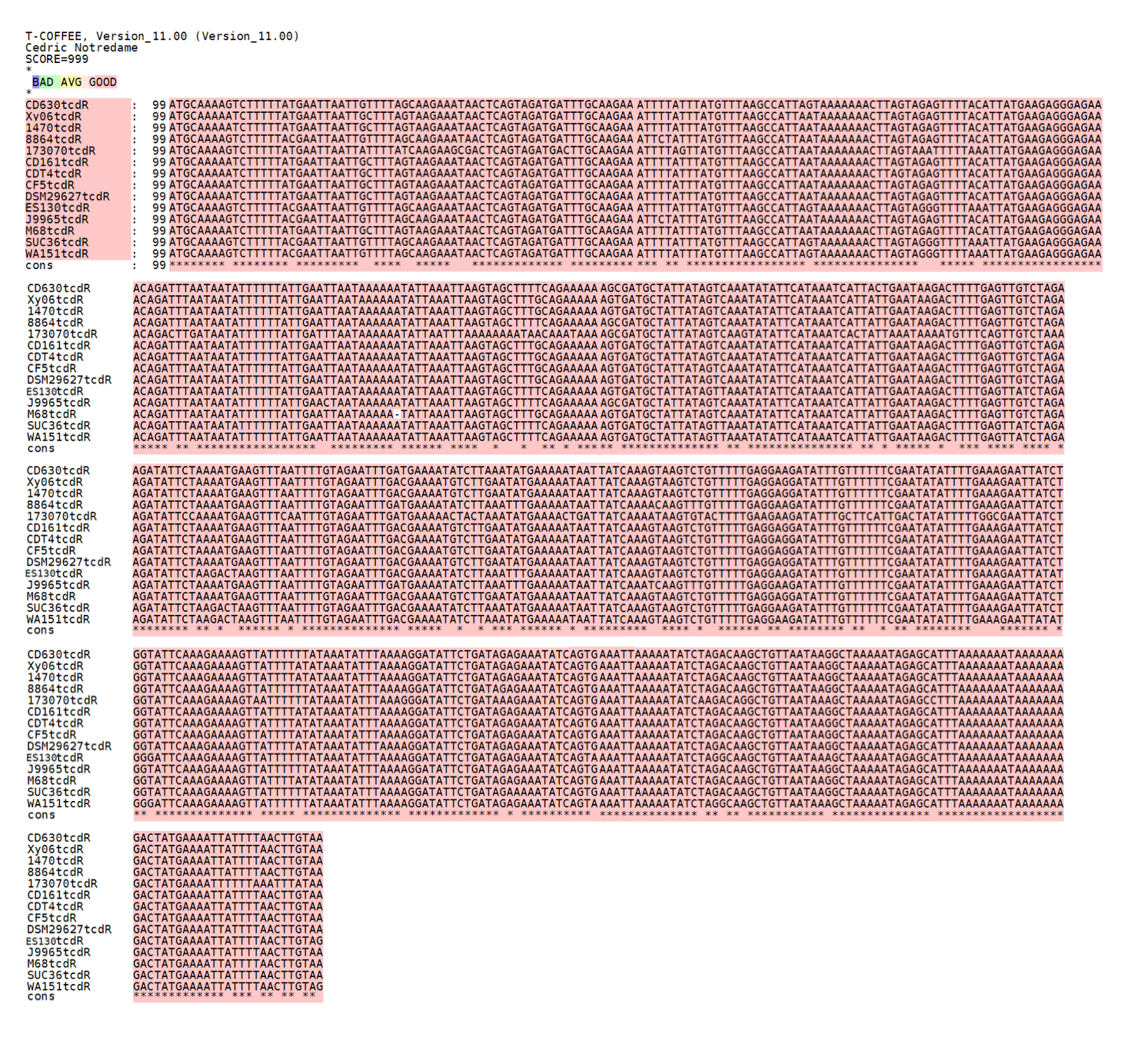
**Supplementary Figure 2. Toxin A-B+ *tcdR* alignment.** T-coffee alignment of *tcdR* nucleotide sequences. Dashes indicate gaps in the alignment, and asterisks (*) in the bottom row indicate conserved (cons) nucleotide sequence. The alignment score is 999 out of 1000 for the total similarity of all sequences, and the comparison of individual sequences to the group is scored out of 100. The highlighted color indicates whether the alignment is of bad (blue), average (yellow), or red (good) quality.


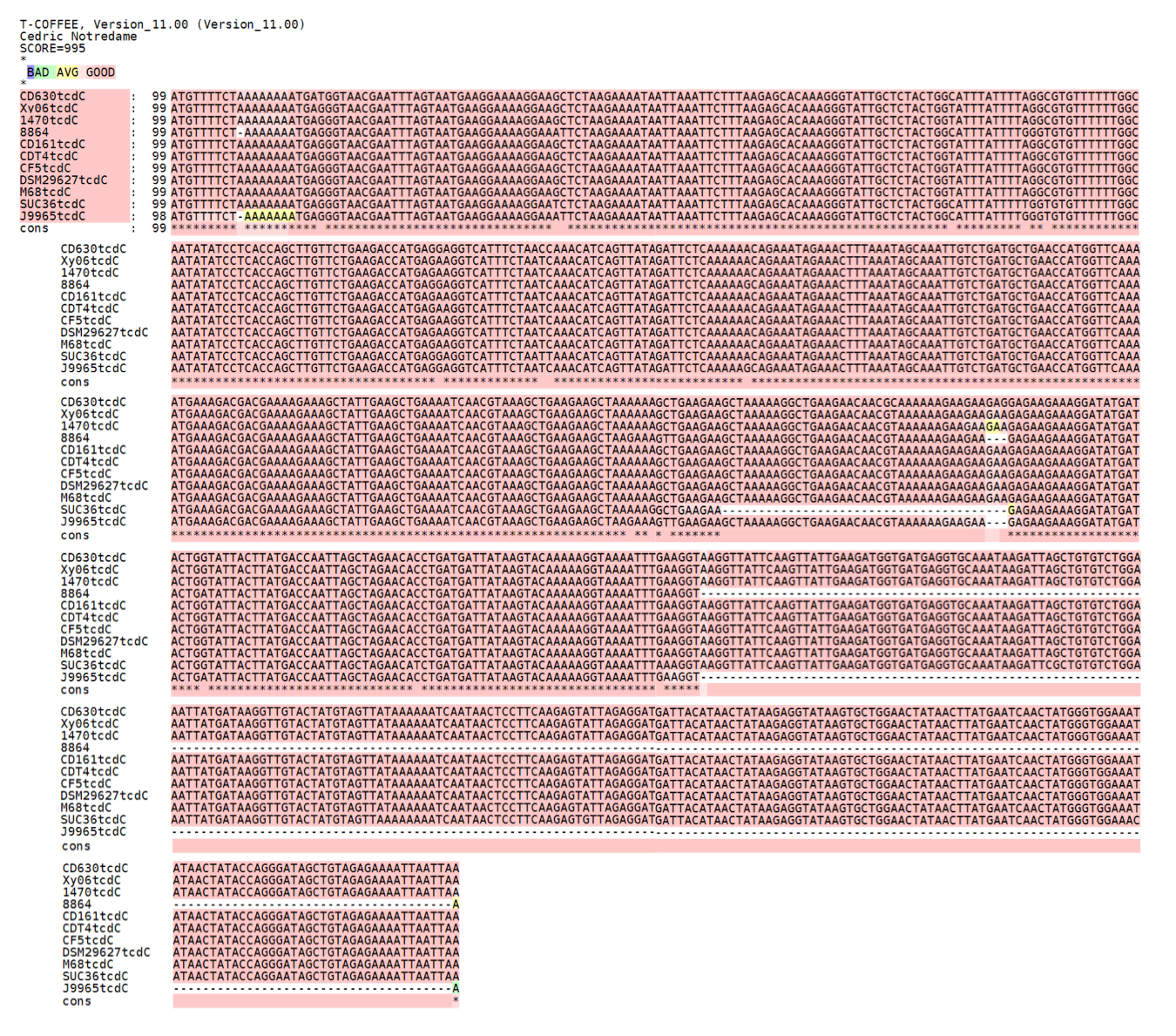


**Supplementary Figure 3. Toxin A-B+ *tcdC* alignment.** T-coffee alignment of *tcdC* nucleotide sequences. Dashes indicate gaps in the alignment, and asterisks (*) in the bottom row indicate conserved (cons) nucleotide sequence. The alignment score is 995 out of 1000 for the total similarity of all sequences, and the comparison of individual sequences to the group is scored out of 100. The highlighted color indicates whether the alignment is of bad (blue), average (yellow), or red (good) quality.

# Supplementary Tables

**Supplementary Table 1**. Sequenced A-B+ *C. difficile* strains (CG- complete genome sequence, WGS- whole genome shotgun sequence, SMRT- single-molecule real-time, ND – not determined).

| **Name** | **Size (bp)** | **GC %** | **CDS** | **tRNAs** | **rRNAs** | **Accession** | **Sequence Type** | **Seqeuncing Technology** | **Isolate location** | **Ribotype (RT)** | **Sequence Type (ST)** |
| --- | --- | --- | --- | --- | --- | --- | --- | --- | --- | --- | --- |
| Xy06 | 4194266 | 28.5 | 3927 | 48 | 11 | NZ_JANFNF000000000.1 | WGS | Illumina | China | 017 | 37 |
| 1470 | 4119431 | 28.4 | 3,710 | 70 | 18 | NZ_OEZL00000000.1 | WGS | Illumina | Belgium | 017 | 37 |
| 8864 | 4051376 | 28.3 | 3,630 | 72 | 26 | NZ_OEZE00000000.1 | WGS | Illumina | UK | 59 | 62 |
| SUC36 | 4149221 | 28.9 | 3,851 | 67 | 27 | NZ_OEZZ00000000.1 | WGS | Illumina | Indonesia | 078 | 195 |
| J9965 | 4298743 | 28.2 | 4,000 | 67 | 31 | NZ_OEZM00000000.1 | WGS | Illumina | Japan | SLO 032 | 194 |
| M68 | 4308325 | 28.9 | 3737 | 109 | 40 | FN668375.1 | CG | Ion Torrent | Ireland | 017 | 37 |
| DSM 29627 | 4209547 | 28.8 | 3,830 | 10 | 35 | CP016102.1 | CG | SMRT, Illumina | France | 017 | 37 |
| aCF5 | 4159517 | 28.5 | 3,684 | 65 | 26 | FN665652.1 | CG | 454 Life Sciences GS-20, 454 Life Sciences GS-FLX, Illumina | USA | 017 | 37 |
| CD161 | 4295210 | 28.9 | 4,090 | 95 | 31 | NZ_CP029154.1 | CG | Illumina, PacBio | China | ND | 37 |
| CDT4 | 4240954 | 28.9 | 3,847 | 90 | 35 | NZ_CP029152.1 | CG | Illumina, PacBio | China | ND | 37 |
| ES130 | 4106157 | 28.0 | 3,758 | 27 | 9 | NZ_OEZV00000000.1 | WGS | Illumina | Australia | SL0 101 | 166 |
| WA151 | 3978952 | 28.2 | 3,648 | 24 | 8 | NZ_OEZY00000000.1 | WGS | Illumina | Australia | SL0 098 | 167 |
| 173070 | 4217797 | 28.4 | 3,866 | 64 | 20 | NZ_OEZH00000000.1 | WGS | Illumina | Spain | 015 | 200 |
| G89 | 4240344 | 29 | 3,883 | 90 | 35 | NZ_CP081021.1 | CG | Illumina, PacBio | China | H, PKI-RT017 | 81 |
| CD060 | 4289668 | 29 | 3,967 | 90 | 35 | NZ_CP081023.1 | CG | Illumina,  PacBio | China | H, PKI-RT017 | 81 |

**Supplementary Table 2**. Putative prophage carriage in Xy06 as determined by PHASTER.

| **Region** | **Length (kb)** | **Position** | **Completeness** | **Score** | **# Total Proteins** | **GC %** |
| --- | --- | --- | --- | --- | --- | --- |
| 1 | 7.8 | 2298-10101 | Incomplete | 50 | 11 | 28.8 |
| 2 | 44.5 | 873016-917522 | Complete | 100 | 43 | 29.4 |
| 3 | 27.3 | 1326839-1354229 | Incomplete | 50 | 9 | 28 |
| 4 | 50 | 1554466-1604510 | Complete | 140 | 60 | 27.8 |
| 5 | 27.3 | 1945106-1972411 | Incomplete | 60 | 30 | 28 |
| 6 | 16.7 | 2184393-2201130 | Incomplete | 30 | 9 | 29.5 |
| 7 | 51.3 | 2284301-2335656 | Incomplete | 40 | 47 | 28.9 |
| 8 | 22.7 | 2945405-2968203 | Incomplete | 50 | 11 | 29.7 |
| 9 | 7.2 | 3390956-3398198 | Incomplete | 30 | 8 | 28.4 |
| 10 | 17.7 | 3459496-3477242 | Incomplete | 40 | 19 | 28.5 |

**Supplementary Table 3.** BLASTn comparisons between transposon sequence queries and the Xy06 genome.

| **Strain** | **Transposon** | **Identity (%)** | **Query Cover (%)** | **E-value** |
| --- | --- | --- | --- | --- |
| CD630 | CTn*1* | 92.04 | 56 | 0 |
|  | CTn*2* | 85.53 | 62 | 0 |
|  | CTn*3* (Tn*5397*) | 88.36 | 69 | 0 |
|  | CTn*4* | No significant similarity | | |
|  | CTn*5* | 99 | 90 | 0 |
|  | CTn*6* | 76.17 | 27 | 0 |
|  | Tn*5398* | 97.11 | 46 | 0 |
| R20291 | Tn*6103* | 98.06 | 42 | 0 |
|  | Tn*6104* | 89.76 | 3 | 0 |
|  | Tn*6105* | No significant similarity | | |
|  | Tn*6106* | 98.23 | 98 | 0 |
| QCD-23M63 | Tn*6073* | 92.08 | 53 | 0 |
|  | Tn*6107* | 92.06 | 50 | 0 |
| QCD-66C26 | Tn*6110* | 98.06 | 62 | 0 |

**Note:** The nucleotide sequence of each transposon was mined from the GenBank annotations of the given strains before performing the analysis online through NCBI (<https://blast.ncbi.nlm.nih.gov/Blast.cgi>).
